# Value of nonenhanced CT combined with laboratory examinations in the diagnosis of acute suppurative cholecystitis treated with percutaneous cholecystostomy: a retrospective study

**DOI:** 10.1186/s12876-022-02224-x

**Published:** 2022-03-29

**Authors:** Bai-Qing Chen, Feng Xie, Guo-Dong Chen, Xue Li, Xue Mao, Bao Jia

**Affiliations:** 1 Department of Nuclear Medicine, The People’s Hospital of Liaoning Province, 33 Wenyi Road, Shenhe District, Shenyang, 110016 China; 2 Dalian Medical University, 9 Western Sections, Lvshun South Street, Lvshunkou District, Dalian, 116044 China; 3 Department of Radiology, Panjin Liaohe Oilfield Gem Flower Hospital, 26 Yingbin Road, Xinglongtai District, Panjin, 124010 China

**Keywords:** Acute cholecystitis, Gallbladder empyema, Percutaneous cholecystostomy, Computed tomography, Logistic regression

## Abstract

**Purposes:**

In this study, we aimed to identify the distribution of presenting laboratory and nonenhanced computed tomography (CT) imaging features within 48 h before percutaneous cholecystostomy (PC) and create a model to appropriately guide the diagnosis of acute suppurative cholecystitis (ASC).

**Methods:**

The study population included 204 acute cholecystitis patients who underwent PC. Based on the timing of the last laboratory and CT examinations before PC, the patients were divided into two groups: within 48 h before PC (Group 1, *n* = 138) and over 48 h before PC (Group 2, *n* = 63). The clinical features of the ASC patients in the two groups were compared. A multivariable model for the diagnosis of ASC in the patients in Group 1 was developed.

**Results:**

Thirty-nine patients in Group 1 had ASC (28.3%). Gallbladder stones, common bile duct stones, gallbladder wall thickness > 2.85 mm, and neutrophil granulocytes > 82.55% were confirmed to be independent risk factors for ASC. The receiver operating characteristic curve of the recurrence prediction model verified its accuracy (area under the curve: 0.803). Compared with the ASC patients in Group 2, the ASC patients in Group 1 had a higher proportion of pericholecystic exudation or fluid (*P* = 0.013) and thicker gallbladder walls (*P* = 0.033).

**Conclusions:**

Using nonenhanced CT imaging features and cutoffs for neutrophil granulocytes, we were able to identify a simple algorithm to discriminate ASC. The degree of local inflammation of the gallbladder in ASC patients progressively increases over time, and these changes can be observed on nonenhanced CT images. However, the symptoms of abdominal pain are of little help in estimating the disease duration in elderly patients.

**Supplementary Information:**

The online version contains supplementary material available at 10.1186/s12876-022-02224-x.

## Introduction

Acute suppurative cholecystitis (acute cholecystitis with gallbladder empyema) is thought to represent one of the most severe forms of acute cholecystitis (AC) [1]. Conventional antibiotic treatment cannot effectively treat acute suppurative cholecystitis (ASC) in most patients, and ASC is a risk factor for gallbladder perforation in patients with AC [2]. The mortality and difficulty grade of laparoscopic cholecystectomy (LC) increase with the development of ASC. ASC is classified into grade 4—the highest difficulty grade—according to the Nassar scale, which is a system for grading the operative difficulty of LC [3]. Therefore, determining whether a patient has progressed to ASC based on their clinical features is of great importance for reducing the mortality and adjusting the treatment strategies for such patients.

If a highly effective diagnosis model could be developed based on common clinical features, it would simultaneously improve the efficiency of treatment and reduce medical expenses. Based on this principle, we chose nonenhanced CT rather than enhanced CT. Many patients present severe symptoms when they see a doctor, and elderly patients also have other complications or underlying illnesses. Therefore, nonenhanced CT is safer for these patients, meets the diagnostic needs of cholecystitis, and is cheaper.

Currently, LC is the main surgical method used to treat for ASC. However, for high-risk patients, percutaneous cholecystostomy (PC) can be used as an alternative treatment to LC [4–6]. LC and PC can both confirm ASC during treatment. PC has a wide range of applications [7–9]. For example, young patients prefer to keep their gallbladders in situ, while some older and frailer patients can only undergo PC because of their poor physical condition. Therefore, it was more reasonable to select patients who underwent PC as the study population.

Considering that ASC is a stage of the progression of AC and in a dynamic process [10], laboratory indicators and imaging data obtained many days before surgery cannot accurately reflect the "real situation" of patients when the disease progresses to ASC. Therefore, we aimed to identify the distribution of presenting laboratory and nonenhanced CT scan imaging features within 48 h before PC and to create a model that appropriately guides the diagnosis of ASC patients.

## Materials and methods

### Patient population

Detailed information regarding the patients was retrieved from the People’s Hospital of Liaoning Province. All procedures performed in studies involving human participants were conducted in accordance with the ethical standards of the institutional and national research committee with the 1964 Declaration of Helsinki and its later amendments or comparable ethical standards. This retrospective study was approved by the Ethics Committee of the People’s Hospital of Liaoning Province and strictly adhered to the tenets of the Declaration of Helsinki (code of Ethical approval for scientific research project: 2021 Ethical Scientific Research Approval No. KS003). Given the retrospective design of this study, a waiver of participant informed consent was granted by the Ethics Committee of the People’s Hospital of Liaoning Province.

The electronic medical database of our institution was searched for records of 316 AC patients who underwent PC for the first time between January 1, 2017, and June 30, 2021. The diagnosis of AC was based on clinical symptoms and signs (fever, abdominal pain, positive sonographic Murphy’s sign, or elevated inflammatory markers, such as white blood cells) and radiologic studies by abdominal US (ultrasonography), CT and so on [11]. Patients with concurrent or secondary pancreatitis (n = 41) and pancreatic trauma (n = 1) were excluded. Among the remaining 274 patients, those with blood in their bile (n = 4), gallbladder mucocele (n = 1) and bile with a small floccule (n = 2) were also excluded. Patients who lacked CT images and laboratory values from our institution were also excluded (n = 63).

**Fig. 1:**
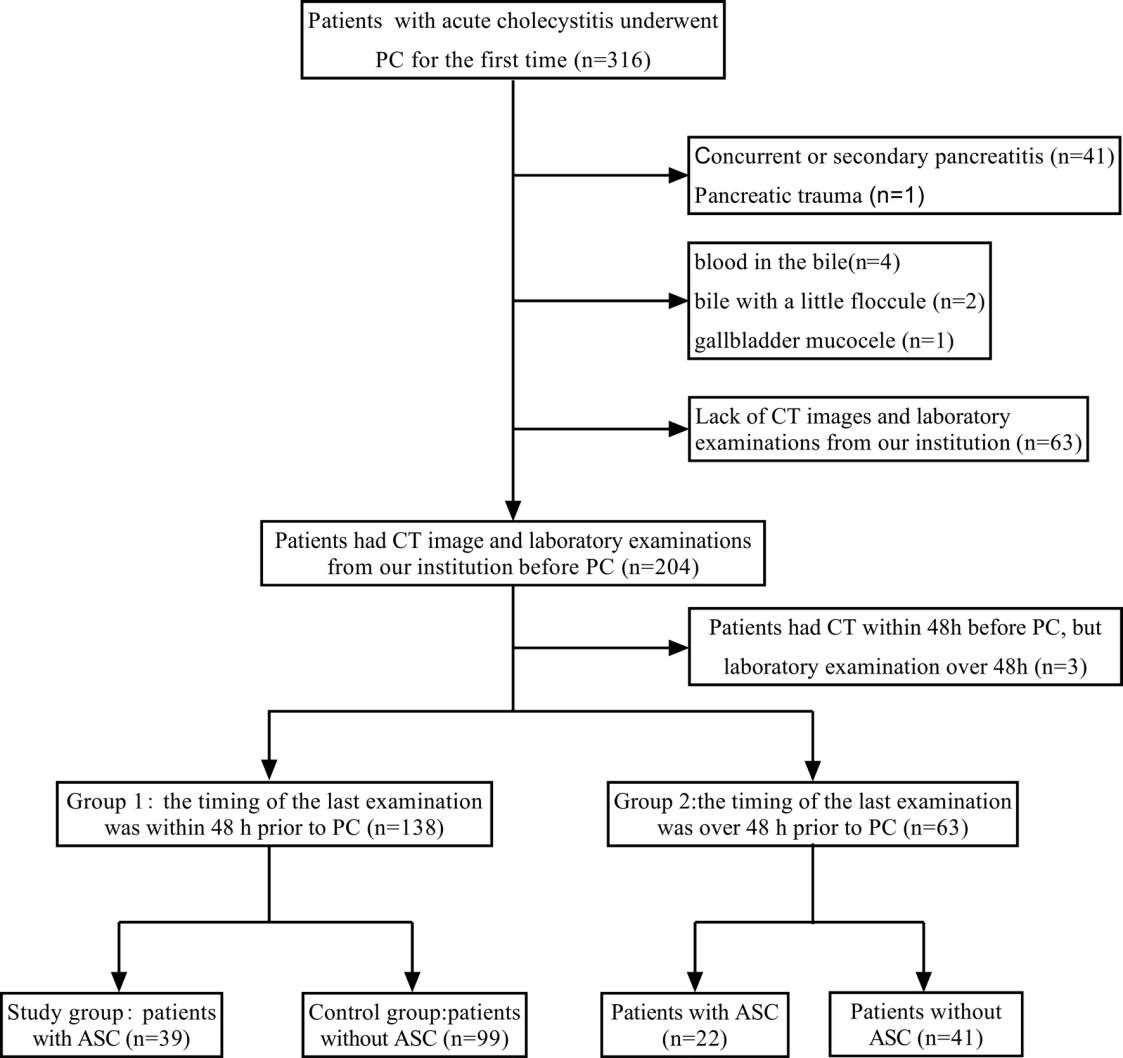
Study flow chart. PC, percutaneous cholecystostomy; ASC, acute suppurative cholecystitis

The diagnostic criteria of ASC were aspirating pus from the gallbladder during PC. This study ultimately included 204 AC patients, including 62 patients with ASC (Fig. 1).

### Percutaneous cholecystostomy and gallbladder contents

All the patients received initial treatment with broad-spectrum antimicrobials. Patients whose condition worsened or failed to clinically improve within 48 h were reevaluated by surgeons, anesthesiologists and interventional radiologists, and a decision was made depending on the patient’s preference for either emergent LC or PC. PC was performed by an experienced interventional radiologist who had 15 years of experience. In brief, using local anesthesia and sterile techniques, a needle was introduced into the gallbladder via the transhepatic or transperitoneal route under ultrasonographic guidance. The gallbladder contents were aspirated via a 10-mL needle. Then, a 0.035-inch guidewire was inserted, followed by placement of an 8-Fr pigtail catheter [11]. The characteristics of the bile were observed during PC.

### Gallbladder CT characteristics

The CT examinations were performed using one of three different scanners (Somatom Definition Flash, Siemens, Germany; Somatom Definition AS+, Siemens, Germany; Aquilion 128, Toshiba, Japan) available at our institution. The scanning parameters varied during the study period and with different scanners: the collimation ranged from 1.25 to 7 mm; the pitch ranged from 0.75 to 1.5; and the section thickness ranged from 1 to 5 mm. For the purpose of this study, we only reviewed the most recent nonenhanced CT scan images obtained before PC. The CT features followed those described in the review by Shakespear et al. [12].

**Fig. 2:**
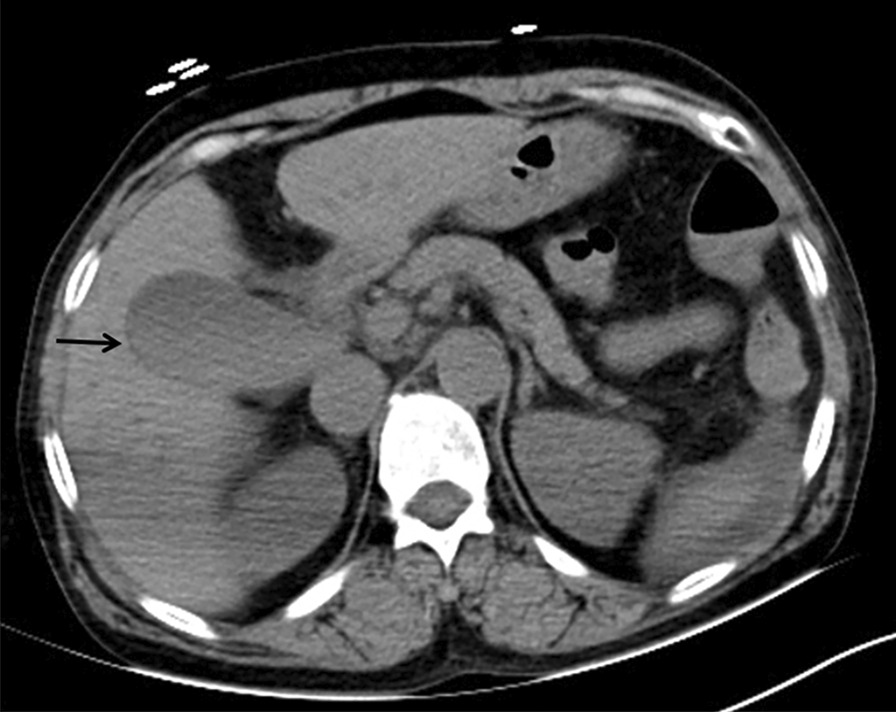
A 54-year-old female with stratification of bile in the lumen. A nonenhanced CT showed stratification of bile (arrow) in the gallbladder. The patient underwent PC the next day, and the bile was black and viscous

**Fig. 3:**
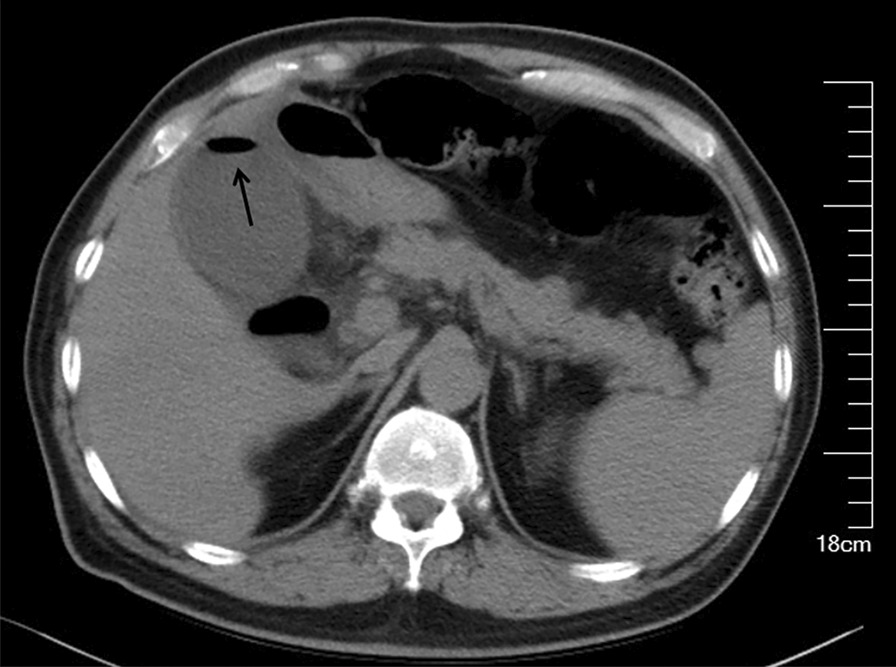
A 59-year-old male with gas within the gallbladder lumen. A nonenhanced CT showed gas (arrow) within the gallbladder lumen. The patient underwent PC on the same day, and the bile was purulent

**Fig. 4:**
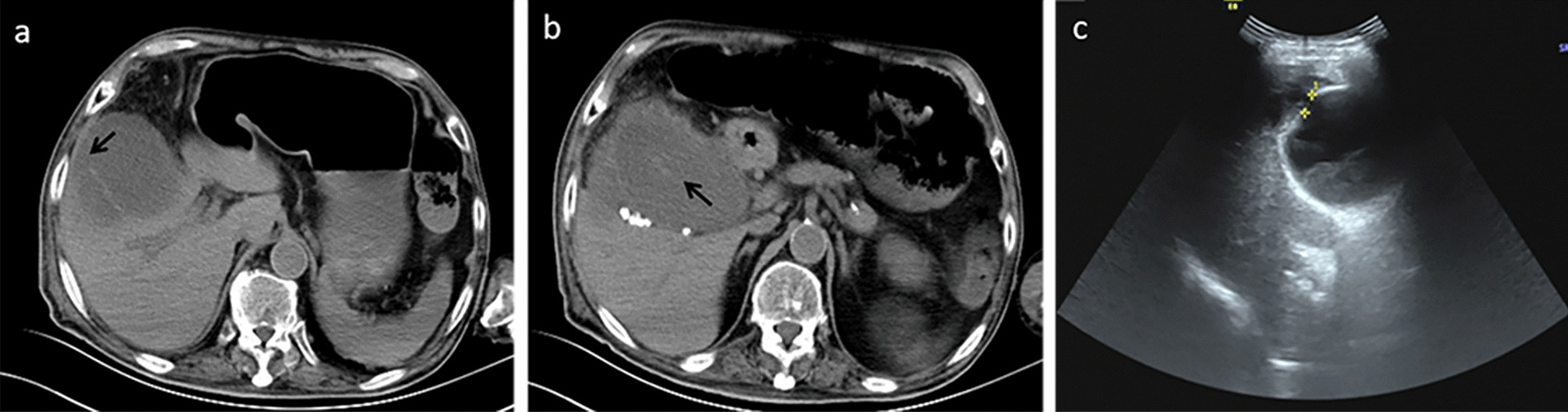
An 86-year-old male with defects in the gallbladder wall. **a** Nonenhanced CT showed defects in the gallbladder wall (arrow). **b** Nonenhanced CT showed a sloughed intraluminal membrane (arrow). **c** US showed defects in the gallbladder wall and a subhepatic hypoechoic area

Two radiologists who were blinded to the radiologic findings, clinical history and laboratory or pathologic findings independently reviewed these imaging data, and they reached an agreement. The following features were recorded: (a) gallbladder stones (cystic duct, neck of the gallbladder, body, or fundus); (b) stratification of bile in the lumen (Fig. 2); (c) gas within the gallbladder lumen (Fig. 3); (d) defects in the gallbladder mucosa, sloughed intraluminal membranes or defects in the gallbladder wall (Fig. 4); (e) thickness of the gallbladder wall (two reviewers measured the three thickest points they found, and then calculated the average value); (f) short axis and long axis of gallbladder; (g) pericholecystic exudation or fluid; (h) hypodense hepatic parenchyma of the gallbladder fossa; and (i) common bile duct (CBD) stones.

### Other variables

We recorded patient baseline data (including age, sex, and ASA score) and preoperative clinical features (including temperature, laboratory values, complications, etc.). The maximum temperature before PC was recorded as the preoperative body temperature. To achieve our goal, the time of the last nonenhanced CT scan and laboratory examination before PC was recorded.

### Statistical analysis

Categorical data are expressed as counts and proportions, and continuous data are expressed as the median and quartile range. The chi-square test or Fisher’s exact test was used to compare the categorical variables, and the Mann–Whitney U test was conducted to compare the continuous variables. The optimum threshold for continuous variables with *P* values ≤ 0.10 in the univariate analysis was determined by identifying the point on the ROC curve with the highest overall accuracy. The continuous variables were transformed to binary categorical variables based on the threshold determined by the ROC analysis. The significant variables based on the univariate analysis (*P* ≤ 0.10) were considered for the multivariate analysis. A forward selection procedure (likelihood ratio) was applied to obtain the final regression model. We developed a diagnostic model using the beta coefficient of the logistic regression model. The diagnostic performance of the model was evaluated according to the receiver operating characteristic curve, and the repeatability of the model was validated by fivefold cross validation. Analyses were performed using SPSS version 24 (IBM, Armonk, NY, USA) and R version 3.6.3 (The R Foundation for Statistical Computing, Vienna, Austria).

## Results

### Clinical features

A total of 204 patients (116 male; range, 31–94 years) who underwent PC were identified. Sixty-two patients had ASC, which was diagnosed following aspiration of pus from the gallbladder. All these patients had gallbladder CT and laboratory data. We collected the data obtained during the most recent nonenhanced CT scan before PC: 80 patients underwent CT on the day of PC, 55 patients underwent CT the day before PC, 30 patients underwent CT 2 days before PC, and 39 patients underwent CT more than 2 days before PC (range, 3–15 days). In summary, 141 patients had CT results that were obtained within 48 h prior to PC, but three of these patients did not undergo laboratory examination within 48 h before PC, i.e., 138 patients underwent both CT and laboratory examination within 48 h before PC.

The patients were divided into two groups based on the timing of the last examination before PC: Group 1, within 48 h before PC, and Group 2, over 48 h before PC. The baseline data of the two groups are provided in Table 1.

**Table 1:** Preoperative clinical characteristics of the patients

Variable	Group 1 (n = 138)	Group 2 (n = 63)	*P* value
Sex [male (%)]	82 (59.4%)	33 (52.4%)	0.349
Age (years)	73.00 (63.00–82.00)	76.00 (63.00–84.00)	0.526
Length of stay before PC (days)	1 (0–3)	5 (3–12)	0.000
ASA score > 2	72 (52.2%)	36 (57.1%)	0.512
Cerebrovascular disease	29 (21.0%)	17 (27.0%)	0.350
Diabetes	42 (30.4%)	15 (23.8%)	0.334
Parenteral nutrition	19 (13.8%)	11 (17.5%)	0.496
Preoperative body temperature (℃)	37.7 (36.7–39.0)	38.4 (37.1–39.0)	0.043
Initial laboratory values			
Platelets (× 10^9^ L)	190.50 (138.25–260.75)	188.00 (157.00–246.00)	0.769
White blood cells (× 10^9^ L)	12.25 (8.30–16.18)	11.35 (7.60–16.95)	0.464
Neutrophil granulocytes (%)	86.30 (79.28–90.73)	83.80 (79.60–89.90)	0.145
ALT (U/L)	30.85 (19.00–66.33)	30.00 (17.00–54.80)	0.632
STB (μmol/L)	23.45 (15.68–40.13)	26.40 (15.70–40.60)	0.834
UCB (μmol/L)	12.95 (7.05–22.03)	13.40 (7.40–24.20)	0.672
Preoperative CT characteristics			
Gallbladder stones	72 (52.2%)	32 (50.8%)	0.856
Cystic duct or neck of the gallbladder stones	56 (40.6%)	19 (30.2%)	0.156
Stratification of bile in the lumen	13 (9.4%)	4 (6.3%)	0.468
Gas within the gallbladder lumen	4 (2.9%)	1 (1.6%)	0.948
Defects in the gallbladder mucosa or sloughed intraluminal membranes	3 (2.2%)	1 (1.6%)	1.000
Defects in the gallbladder wall	1 (0.7%)	0 (0%)	1.000
Pericholecystic exudation or fluid	77 (55.8%)	27 (42.9%)	0.089
Hepatic parenchyma of the gallbladder fossa appeared hypodense	7 (5.1%)	1 (1.6%)	0.433
CBD stones	11 (8.0%)	8 (12.7%)	0.288
Gallbladder wall thickness (mm)	2.85 (2.40–3.50)	2.80 (2.40–3.40)	0.649
Ratio of short to long axis of gallbladder	0.49 (0.43–0.56)	0.46 (0.41–0.54)	0.123
Acute suppurative cholecystitis	39 (28.3%)	22 (34.9%)	0.341

ASA, American Society of Anesthesiologists; ALT, alanine aminotransferase; STB, serum total bilirubin; UCB, unconjugated bilirubin

### The value of combining CT with laboratory values in the diagnosis of ASC

Group 1 was divided into two groups based on the presence or absence of gallbladder empyema. The study group included patients with gallbladder empyema (n = 39), while the control group included patients without gallbladder empyema (n = 99). The clinical features of both groups were compared (Table 2). The study group had a higher proportion (71.8%) of gallbladder stones (*P* = 0.004), the proportion of cystic duct/neck gallbladder stones was higher in the study group (*P* = 0.017), and the gallbladder walls were thicker in the study group (*P* = 0.002).

**Table 2:** Clinical features of Group 1

Variable	Study group (n = 39)	Control group (n = 99)	OR	95% CI	*P* value
Sex [male (%)]	27 (69.2%)	55 (55.6%)	1.800	0.819–3.955	0.141
Age (years)	76.0 (67.0–84.0)	72.0 (63.0–81.0)	–	–	0.110
ASA score > 2	18 (46.2%)	54 (54.5%)	0.714	0.340–1.502	0.374
Cerebrovascular disease	5 (12.8%)	24 (24.2%)	0.460	0.162–1.307	0.138
Diabetes	12 (30.8%)	30 (30.3%)	1.022	0.458–2.284	0.957
Preoperative body temperature (℃)	38.30 (36.80–39.00)	37.40 (36.70–39.00)	–	–	0.365
Initial laboratory values					
Platelets (× 10^9^ L)	164.00 (142.00–243.00)	203.00 (133.00–275.00)	–	–	0.193
White blood cells (× 10^9^ L)	12.57 (8.95–16.42)	12.10 (8.23–16.16)	–	–	0.657
Neutrophil granulocytes (%)	88.90 (84.60–91.20)	84.80 (78.30–90.40)	–	–	0.018
ALT (U/L)	30.90 (21.00–68.00)	30.80 (19.00–63.00)	–	–	0.610
STB (μmol/L)	28.00 (19.70–60.00)	22.80 (13.70–35.70)	–	–	0.079
UCB (μmol/L)	16.20 (7.10–27.40)	12.40 (6.90–19.50)	–	–	0.107
Preoperative CT characteristics					
Gallbladder stones	28 (71.8%)	44 (44.4%)	3.182	1.427–7.097	0.004
Cystic duct or neck of the gallbladder stones	22 (56.4%)	34 (34.3%)	2.474	1.161–5.273	0.017
Stratification of bile in the lumen	1 (2.6%)	12 (12.1%)	0.191	0.024–1.520	0.159
Gas within the gallbladder lumen	3 (7.7%)	1 (1.0%)	8.167	0.823–81.064	0.123
Defects in the gallbladder mucosa or sloughed intraluminal membranes	2 (5.1%)	1 (1.0%)	5.297	0.466–60.175	0.193
Defects in the gallbladder wall	1 (2.6%)	0 (0%)	–	–	0.283
Pericholecystic exudation or fluid	27 (69.2%)	50 (50.5%)	2.205	1.005–4.839	0.046
Hepatic parenchyma of the gallbladder fossa appeared hypodense	3 (7.7%)	4 (4.0%)	1.979	0.422–9.281	0.653
CBD stones	7 (17.9%)	4 (4.0%)	5.195	1.427–18.914	0.018
Gallbladder wall thickness (mm)	3.10 (2.80–3.80)	2.70 (2.40–3.40)	–	–	0.002
Ratio of short to long axis of gallbladder	0.49 (0.45–0.56)	0.48 (0.43–0.56)	–	–	0.524

ASA, American Society of Anesthesiologists; ALT, alanine aminotransferase; STB, serum total bilirubin; UCB, unconjugated bilirubin

CT images of pericholecystic exudation or fluid (*P* = 0.046) and CBD stones (*P* = 0.018) were recorded in significantly more patients in the study group than in the control group. The percentage of neutrophil granulocytes was significantly higher in the study group (*P* = 0.018). There was no significant difference in the proportion of patients with an ASA score > 2 between the two groups (*P* = 0.374).

In the univariable analysis, gallbladder stones were more influential than the cystic duct or neck of the gallbladder stones. The gallbladder stones included stones in the cystic duct or neck of the gallbladder. Therefore, the latter was not included in the multivariate analysis. The optimum thresholds for preoperative neutrophil granulocytes, preoperative serum total bilirubin (STB), and gallbladder wall thickness were determined by identifying the point on the receiver operating characteristic (ROC) curve with the highest overall accuracy. These continuous variables were transformed to binary categorical variables based on the thresholds determined by ROC analysis.

**Fig. 5:**
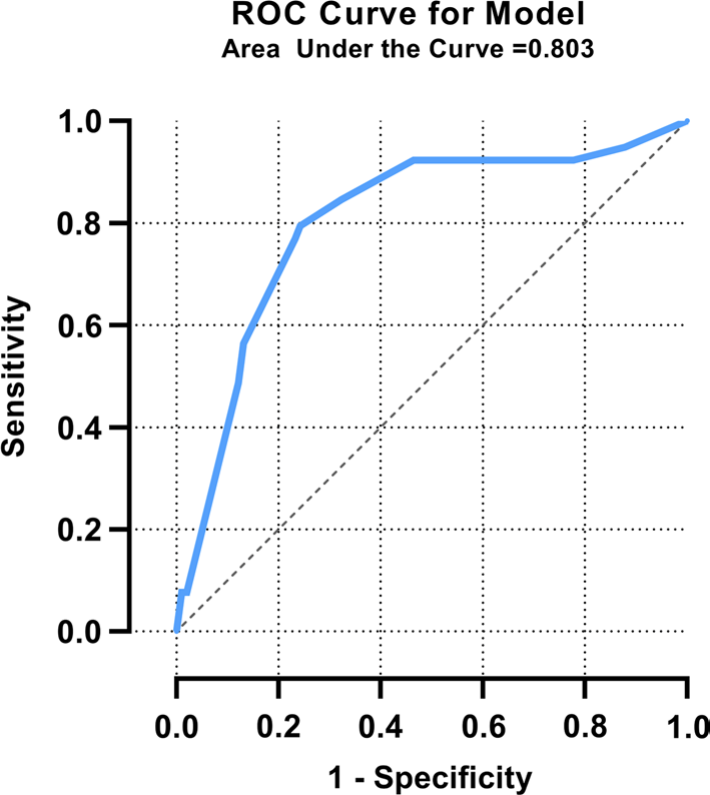
Receiver operating characteristic (ROC) curve of the diagnostic model

In the multivariate analysis (Table 3), gallbladder stones (OR: 2.706; 95% confidence interval [CI] 1.125–6.512; *P* = 0.026), CBD stones (OR: 4.346; 95% CI 0.998–18.921; *P* = 0.050), gallbladder wall thickness > 2.85 mm (OR: 4.645; 95% CI 1.860–11.601; *P* = 0.001), and neutrophil granulocytes > 82.55% (OR: 3.804; 95% CI 1.275–11.344; *P* = 0.017) had an effect on the diagnosis of ASC in the study population (Group 1). The logistic regression model was established:$Logit\left( P \right) = - 3.554 + 0.996A + 1.469B + 1.536C + 1.336D$, where A is gallbladder stones (0 if no and 1 if yes), B is CBD stones (0 if no and 1 if yes), C is gallbladder wall thickness > 2.85 mm (0 if no and 1 if yes), and D is neutrophil granulocytes > 82.55 (0 if no and 1 if yes). As shown in Fig. 5, the area under the curve and the 95% CI of the diagnostic model were as follows: area under the curve: 0.803; 95% CI 0.718–0.889.

**Table 3:** Multivariate analysis of risk factors for ASC

Variable	OR	95% CI	*P* value
Gallbladder stones	2.706	1.125–6.512	0.026
CBD stones	4.346	0.998–18.921	0.050
Gallbladder wall thickness (> 2.85 mm)	4.645	1.860–11.601	0.001
Neutrophil granulocytes (> 82.55%)	3.804	1.275–11.344	0.017

CBD, common bile duct; CI, confidence interval; OR, odds ratio

The maximum Youden index was 0.553, corresponding to an optimal cutoff value of − 0.8855. The sensitivity and specificity were 79.5% and 75.8%, respectively. The obtained model was internally validated (fivefold cross-validation), and the mean value of the C-statistics was 0.8021, with a minimum value of 0.7083 and a maximum value of 0.9091.

### Comparison of preoperative clinical features of ASC patients in Group 1 and Group 2

Compared with the ASC patients in Group 2, the ASC patients in Group 1 had a higher proportion of pericholecystic exudation or fluid (36.4% vs. 69.2%, *P* = 0.013) and thicker gallbladder walls (*P* = 0.033) (Additional file 1: Table S1).

### Effect of time on the progression of AC disease

**Fig. 6:**
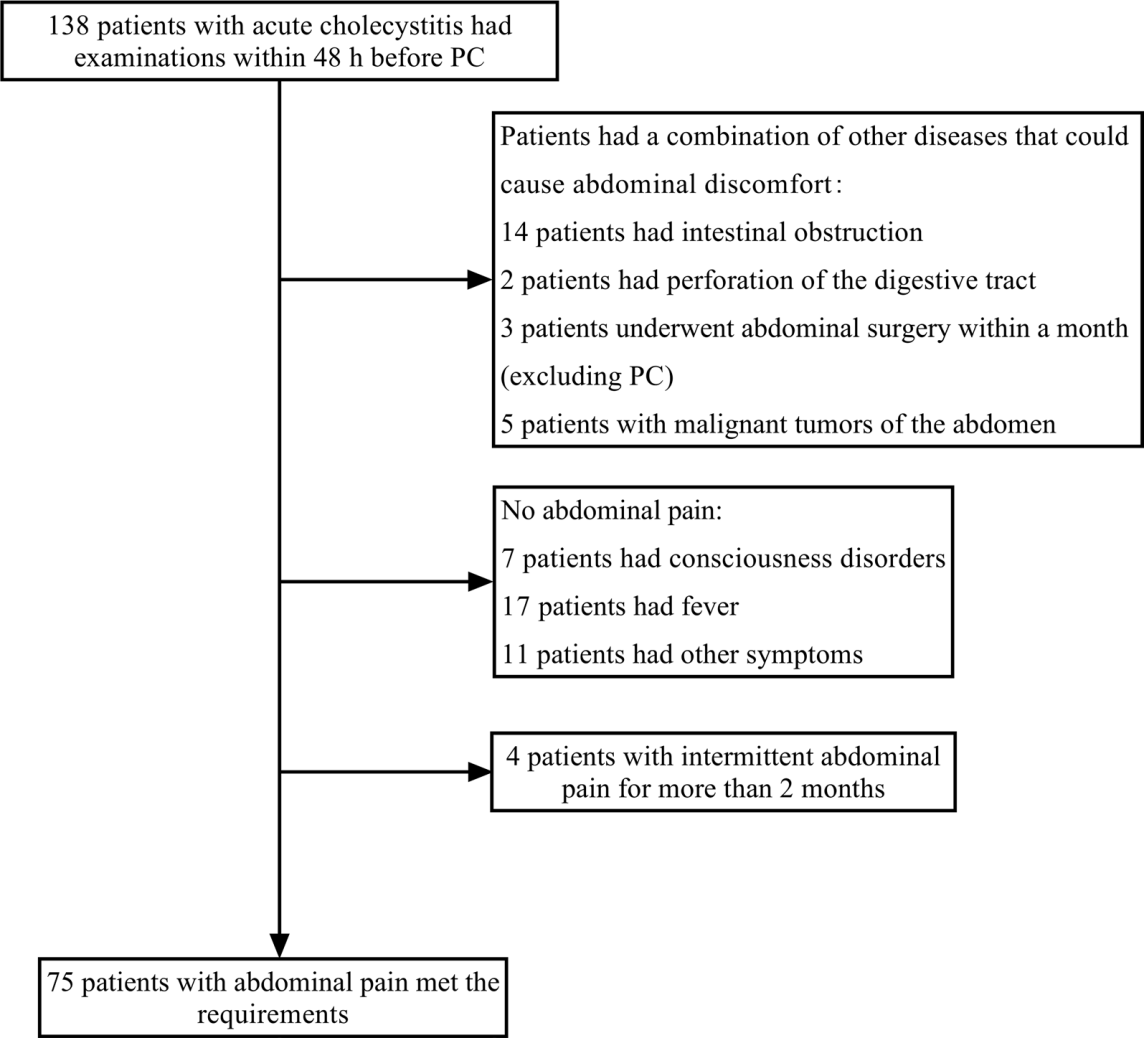
Screening of acute cholecystitis patients with complaints of abdominal pain in Group 1. PC, percutaneous cholecystostomy

We screened AC patients with complaints of abdominal pain in Group 1 (Fig. 6). A total of 75 patients met the requirements, including 25 ASC patients. In patients with ASC, the median time from the first onset of abdominal pain to PC was 3 days (interquartile range, 2–5 days). In patients without ASC, the median time was also 3 days (interquartile range, 2–5 days). There was no significant difference between these groups (*P* = 0.739).

## Discussion

The incidence of ASC was 30.4% in our study. The diagnostic criterion of ASC was the aspiration of pus from the gallbladder during PC. Multivariate analysis revealed that among the factors included in this study, gallbladder stones, CBD stones, gallbladder wall thickness > 2.85 mm, and neutrophil granulocytes > 82.55% were independent risk factors for the diagnosis of ASC.

In our study, gallbladder stones included stones in the cystic duct or neck of the gallbladder and in other locations within the gallbladder. Considering that the pathogenesis underlying 90% of ASC cases is obstruction of the cystic duct by gallstones [13], we evaluated this factor using univariate analysis. However, gallbladder stones were more influential than stones in the cystic duct or neck of the gallbladder. Therefore, when CT images showed that the gallbladder stones were not located in the gallbladder neck or cystic duct, we inferred that there may be undetectable stones obstructing other locations in the biliary system because the stones were too small or were not seen on CT due to their composition.

Eleven patients had CBD stones visible on CT, including three patients without gallbladder stones. One of these 3 patients was diagnosed as ASC. It is a fact that only CBD stones can also cause ASC. When stones obstruct CBD, bile accumulates in the biliary system. As the space of the intrahepatic bile duct is depleted, more bile flows into the gallbladder, eventually leading to ASC development. And when it will progress to ASC may be related to the volume of bile that can be held in the biliary system beyond the gallbladder. The study by El et al. also showed a high prevalence of CBD stones associated with ASC, i.e., 22% of ASC patients were found to have CBD stones with intraoperative cholangiography [5].

In the multivariate analysis, gallbladder stones and CBD stones were the causes of ASC, while gallbladder wall thickness and neutrophil granulocytes reflected the severity of inflammation in AC. Local and systemic inflammation is usually more severe in ASC than in edematous cholecystitis. An increased neutrophil proportion can indicate the presence of infection by pyogenic bacteria, whereas the gallbladder wall thickens with inflammation.

Time affects the changes in inflammation observed in AC, as evidenced by our results. We compared the clinical characteristics of the ASC patients in Group 1 with those of the ASC patients in Group 2. The baseline and proportion of stones were comparable between the two groups of ASC patients, i.e., there was no significant difference in the underlying causative factors between the two groups of ASC patients. However, compared with the ASC patients in Group 2, the ASC patients in Group 1 had a higher proportion of pericholecystic exudation or fluid (36.4% vs. 69.2%, *P* = 0.013) and thicker gallbladder walls (*P* = 0.033). This suggests that the degree of local gallbladder inflammation in these patients progressively increases over time, and these changes can be observed on nonenhanced CT images. However, the median time from the first onset of abdominal pain to PC was 3 days in both the ASC and non-ASC groups (*P* = 0.739). Furthermore, Adachi et al*.* noted that AC progresses to its purulent phase from 7 to 10 days after gallbladder stones obstruct the cystic duct or neck of the gallbladder [10]. This suggests that the duration of abdominal pain symptoms is of little help in estimating the degree of progression of AC in elderly patients. In our experience, in these patients, inappetence and nausea seem to appear earlier than abdominal pain. However, these symptoms are not typical enough, and they do not attract the attention of patients early in the course of disease. Therefore, it is important to look for more appropriate symptoms to more accurately determine the duration of the disease.

Ambe et al. reported a study that included the largest series of ASC patients to date [1], but detailed information about sonographic diagnosis and the time interval between LC and imaging examination were not available in the database used in their study, which made it impossible to consider the characteristics of imaging in ASC patients. Their study showed that male sex, advanced age, ASA score > 2, elevated white blood count (> 12,000/mm^3^) and fever were confirmed as risk factors for ASC. However, our study did not obtain similar conclusions. The differences in results may be related to the different study populations selected. Taking this into consideration, we performed a limited comparison with ASC patients diagnosed during cholecystectomy versus those diagnosed during PC [1]. However, the incidence of ASC in the study by Ambe was not significantly different from the incidence in our study (26.6% vs. 30.4%, *P* = 0.222), and the mean age of the ASC patients in this study was not significantly different from the mean age in the study by Ambe (67.2 years vs. 72.8 years).

LC is the gold standard treatment for AC. However, PC has traditionally been the drainage option of choice for patients who are not surgical candidates [14]. PC is an alternative approach for high-risk patients for whom urgent surgery is contraindicated, such as elderly individuals, critically ill patients, or patients with severe comorbidities. Compared with LC, the role of PC in the management of AC makes it more advantageous in studies on ASC. On the one hand, the presence of concomitant choledocholithiasis at the time of cholecystectomy is an independent predictor of CBD injury [15]. Therefore, many institutions choose to remove CBD stones before LC, but the time between the two procedures can underestimate the significance of CBD stones in predicting ASC. In contrast, CBD stones are removed after PC for high-risk AC patients who are in urgent need of surgical intervention. On the other hand, because the treatment of PC can interfere with the assessment of ASC by LC, patients who underwent PC before LC were excluded from studies that assessed ASC by LC [4, 5]. Patients who underwent PC before LC often had a higher severity of AC and poorer physical status.

Although PC has traditionally been the drainage option of choice for patients who are not candidates for surgery, the rate of adverse events (AEs) could reach 14% and the presence of external drainage may reduce patient quality of life. Currently, endoscopic gallbladder drainage could be considered a possible alternative approach [14]. Endoscopic transpapillary gallbladder drainage (ETGBD) and transmural endoscopic ultrasound-guided gallbladder drainage (EUS-GBD) are two common approaches for endoscopic gallbladder drainage. Of these drainage options, EUS-GBD results a lower incidence of AE and has great potential in the treatment of gallbladder diseases [16]. Raiter et al. reported a case of ASC that was successfully treated with EUS-GBD and used cholecystoscopy through a lumen of the previously implanted lumen-apposing metal stent (LAMS) to treat the cholelithiasis [17]. Mangiavillano et al. also reported clinical success was achieved in treating AC patients with EUS-guided LAMS [18].

There are many strengths to the present study. It is one of the largest retrospective studies evaluating ASC in AC patients treated with PC with an emphasis on using nearly universally available laboratory testing and nonenhanced CT imaging for its diagnostic model. This study also confirmed that examinations within 48 h before surgery were more valuable for the diagnosis of ASC.

There are a few limitations to our study. First, the nature of this retrospectively designed single-center study limits its clinical value. Second, the number of patients in our institution who underwent examinations both within and over 48 h before PC was so small that it is not possible to compare the differences between the results obtained in these two periods in the same group of patients. Third, although PC is more widely performed, the choice of using either PC or LC to treat AC is driven by physician preference, practice patterns, and institutional guidelines. The age distribution of AC patients undergoing PC may vary in different institutions.

In conclusion, using nonenhanced CT imaging features and cutoffs for neutrophil granulocytes, we were able to identify a simple algorithm to discriminate ASC. The degree of local gallbladder inflammation in ASC patients progressively increases over time, and these changes can be observed on nonenhanced CT images. However, the symptoms of abdominal pain are of little help for estimating the disease duration in elderly patients.

## Supplementary Information

**Additional file 1:** Comparison of clinical features of ASC patients in Group 1 and Group 2.

## Data Availability

The datasets used and/or analyzed during the current study are available from the corresponding author on reasonable request. The data are not publicly available due privacy and ethical concerns by the Ethics Committee of the People’s Hospital of Liaoning Province.

## Abbreviations

CT: Computed tomography
PC: Percutaneous cholecystostomy
ASC: Acute suppurative cholecystitis
CBD: Common bile duct
AC: Acute cholecystitis
LC: Laparoscopic cholecystectomy
ASA: American Society of Anesthesiologists
STB: Serum total bilirubin
ROC: Receiver operating characteristic
ALT: Alanine aminotransferase
UCB: Unconjugated bilirubin
CI: Confidence interval
OR: Odds ratio
AE: Adverse event
ETGBD: Endoscopic transpapillary gallbladder drainage
EUS-GBD: Transmural endoscopic ultrasound-guided gallbladder drainage
LAMS: Lumen-apposing metal stent

## References

[1] Ambe PC, Jansen S, Macher-Heidrich S, Zirngibl H (2016). Surgical management of empyematous cholecystitis: a register study of over 12,000 cases from a regional quality control database in Germany. Surg Endosc.

[2] Jansen S, Stodolski M, Zirngibl H, Godde D, Ambe PC (2018). Advanced gallbladder inflammation is a risk factor for gallbladder perforation in patients with acute cholecystitis. World J Emerg Surg WJES.

[3] Griffiths EA, Hodson J, Vohra RS, Marriott P, Katbeh T, Zino S, Nassar AHM (2019). West Midlands Research C: utilisation of an operative difficulty grading scale for laparoscopic cholecystectomy. Surg Endosc.

[4] Kwon YJ, Ahn BK, Park HK, Lee KS, Lee KG (2013). What is the optimal time for laparoscopic cholecystectomy in gallbladder empyema?. Surg Endosc.

[5] El Zanati H, Nassar AHM, Zino S, Katbeh T, Ng HJ, Abdellatif A (2020). Gall bladder empyema: early cholecystectomy during the index admission improves outcomes. JSLS J Soc Laparoendosc Surg.

[6] Mori Y, Itoi T, Baron TH, Takada T, Strasberg SM, Pitt HA, Ukai T, Shikata S, Noguchi Y, Teoh AYB (2018). Tokyo guidelines 2018: management strategies for gallbladder drainage in patients with acute cholecystitis (with videos). J Hepatobiliary Pancreat Sci.

[7] Okamoto K, Suzuki K, Takada T, Strasberg SM, Asbun HJ, Endo I, Iwashita Y, Hibi T, Pitt HA, Umezawa A (2018). Tokyo guidelines 2018: flowchart for the management of acute cholecystitis. J Hepatobiliary Pancreat Sci.

[8] Wang CH, Wu CY, Lien WC, Liu KL, Wang HP, Wu YM, Chen SC (2019). Early percutaneous cholecystostomy versus antibiotic treatment for mild and moderate acute cholecystitis: a retrospective cohort study. J Formosan Med Assoc Taiwan yi zhi.

[9] Yao P, Chang Z, Liu Z (2021). Factors influencing failure to undergo interval cholecystectomy after percutaneous cholecystostomy among patients with acute cholecystitis: a retrospective study. BMC Gastroenterol.

[10] Adachi T, Eguchi S, Muto Y (2021). Pathophysiology and pathology of acute cholecystitis: a secondary publication of the Japanese version from 1992. J Hepatobiliary Pancreat Sci.

[11] Chen BQ, Chen GD, Xie F, Li X, Mao X, Jia B (2021). Percutaneous cholecystostomy as a definitive treatment for moderate and severe acute acalculous cholecystitis: a retrospective observational study. BMC Surg.

[12] Shakespear JS, Shaaban AM, Rezvani M (2010). CT findings of acute cholecystitis and its complications. AJR Am J Roentgenol.

[13] Cai HJ, Wang W, Fang JH, Chen CH, Kong FL, Xu CK (2021). Treatment of acute suppurative cholecystitis with coagulopathy by percutaneous transhepatic gallbladder drainage after hepatic needle-track ablation: report of a new technique. Quant Imaging Med Surg.

[14] Fugazza A, Colombo M, Repici A, Anderloni A (2020). Endoscopic ultrasound-guided gallbladder drainage: current perspectives. Clin Exp Gastroenterol.

[15] Chisholm PR, Patel AH, Law RJ, Schulman AR, Bedi AO, Kwon RS, Wamsteker EJ, Anderson MA, Elta GH, Govani SM (2019). Preoperative predictors of choledocholithiasis in patients presenting with acute calculous cholecystitis. Gastrointest Endosc.

[16] Crino SF, Rimbas M, Gabbrielli A, Larghi A (2019). Endoscopic ultrasound guided gallbladder interventions: a review of the current literature. J Gastrointest Liver Dis JGLD.

[17] Raiter A, Szełemej J, Kozłowska-Petriczko K, Petriczko J, Pawlak KM (2021). The complex advanced endoscopic approach in the treatment of choledocholitiasis and empyema of gallbladder. Endoscopy.

[18] Mangiavillano B, Moon JH, Crino SF, Larghi A, Pham KD, Teoh AYB, Paduano D, Lee YN, Yoo HW, Shin IS (2022). Safety and efficacy of a novel electrocautery-enhanced lumen-apposing metal stent in interventional EUS procedures (with video). Gastrointest Endosc.

