# Supplementary material for: Value of nonenhanced CT combined with laboratory examinations in the diagnosis of acute suppurative cholecystitis treated with percutaneous cholecystostomy: a retrospective study
Source: BMC Gastroenterol. 2022 Mar 29;22:155. doi: 10.1186/s12876-022-02224-x (PMC8966294; doi:10.1186/s12876-022-02224-x)
Supplement: Supplementary file 1 — Additional file 1: Comparison of clinical features of ASC patients in Group 1 and Group 2. [file 12876_2022_2224_MOESM1_ESM.docx]

**Supplementary Table 1 Comparison of clinical features of ASC patients in Group 1 and Group 2**

| Variable | Group 1（n=39） | Group 2（n=22） | *P*-value |
| --- | --- | --- | --- |
| Sex [male (%)] | 27（69.2%） | 14（63.6%） | 0.655 |
| Age (years) | 76.00（67.00–84.00） | 70.00（60.25–87.00） | 0.775 |
| ASA score >2 | 18（46.2%） | 11（50.0%） | 0.773 |
| Cerebrovascular disease | 5（12.8%） | 7（31.8%） | 0.145 |
| Diabetes | 12（30.8%） | 4（18.2%） | 0.283 |
| Parenteral nutrition | 2（5.1%） | 2（9.1%） | 0.951 |
| Preoperative body temperature (℃) | 38.30（36.80–39.00） | 38.15（37.98–39.00） | 0.573 |
| Initial laboratory values |  |  |  |
| Platelets (×10^9^ L) | 164.0（142.0–243.0） | 168.00（99.50–200.50） | 0.476 |
| White blood cells (×10^9^ L) | 12.57（8.95–16.42） | 11.75（8.39–16.29） | 0.658 |
| Neutrophil granulocytes (%) | 88.90（84.60–91.20） | 85.45（79.33–90.43） | 0.174 |
| ALT(U/L) | 30.90（21.00–68.00） | 34.75（20.78–64.95） | 0.898 |
| STB (μmol/L) | 28.00（19.70–60.00） | 31.60（22.85–43.38） | 0.916 |
| UCB (μmol/L) | 16.20（7.10–27.40） | 18.10（11.33–25.95） | 0.680 |
| Preoperative CT characteristics |  |  |  |
| Gallbladder stones | 28（71.8%） | 16（72.7%） | 0.938 |
| Stratification of bile in the lumen | 1（2.6%） | 1（4.5%） | 1.000 |
| Gas within the gallbladder lumen | 3（7.7%） | 1（4.5%） | 1.000 |
| Defects in the gallbladder mucosa or sloughed intraluminal membranes | 2（5.1%） | 0（0.0%） | 0.531 |
| Defects in the gallbladder wall | 1（2.6%） | 0（0.0%） | 1.000 |
| Pericholecystic exudation or fluid | 27（69.2%） | 8（36.4%） | 0.013 |
| Hepatic parenchyma of the gallbladder fossa appeared hypodense | 3（7.7%） | 1（4.5%） | 1.000 |
| CBD stones | 7（17.9%） | 4（18.2%） | 1.000 |
| The thickness of gallbladder wall（mm） | 3.10（2.80–3.80） | 2.90（2.40–3.30） | 0.033 |
| Ratio of short to long axis of gallbladder | 0.49（0.45–0.56） | 0.46（0.41–0.55） | 0.169 |
| ASA, American Society of Anesthesiologists; ALT, alanine aminotransferase; STB, serum total bilirubin; UCB, unconjugated bilirubin | | | |
